# Generative Models and Sentence Transformers for the Recognition and Normalization of Continuous and Discontinuous Phenotype Mentions: Model Development and Evaluation

**DOI:** 10.2196/68558

**Published:** 2025-11-05

**Authors:** Areej Alhassan, Viktor Schlegel, Monira Aloud, Riza Batista-Navarro, Goran Nenadic

**Affiliations:** 1 Department of Computer Science School of Engineering University of Manchester Manchester United Kingdom; 2 Department of Management Information Systems College of Business Administration King Saud University Riyadh Saudi Arabia; 3 Imperial Global Singapore Imperial College London Singapore Singapore

**Keywords:** named entity recognition, discontinuous NER, human phenotype ontology, named entity normalization, LLM, sentence transformers, clinical information extraction

## Abstract

**Background:**

Extracting genetic phenotype mentions from clinical reports and normalizing them to standardized concepts within the human phenotype ontology are essential for consistent interpretation and representation of genetic conditions. This is particularly important in fields such as dysmorphology and plays a key role in advancing personalized health care. However, modern clinical named entity recognition methods face challenges in accurately identifying discontinuous mentions (ie, entity spans that are interrupted by unrelated words), which can be found in these clinical reports.

**Objective:**

This study aims to develop a system that can accurately extract and normalize genetic phenotypes, specifically from physical examination reports related to dysmorphology assessment. These mentions appear in both continuous and discontinuous lexical forms, with a focus on addressing challenging discontinuous entity spans.

**Methods:**

We introduce DiscHPO, a 2-phase pipeline consisting of a sequence-to-sequence named entity recognition model for span extraction, and an entity normalizer that uses a sentence transformer biencoder for candidate generation and a cross-encoder reranker for selecting the best candidate as the normalized concept. This system was tested as part of our participation in Track 3 of the BioCreative VIII shared task.

**Results:**

For overall performance on the test set, the top-performing model for entity normalization achieved an *F*_1_-score of 0.723, while the best span extraction model reached an *F*_1_-score of 0.665. Both scores surpassed those of 2 baseline models using the same dataset, indicating superior efficacy in handling both continuous and discontinuous spans. On the validation set, we were able to demonstrate our system’s ability to recognize these mentions, with the model achieving an *F*_1_-score of 0.631 for exact match on discontinuous spans only.

**Conclusions:**

The findings suggest that exact extraction of entity spans may not always be necessary for successful normalization. Partial mention matches can be sufficient as long as they capture the essential concept information, supporting the system’s utility in clinical downstream tasks.

## Introduction

### Overview

Information extraction (IE) from unstructured text is a crucial task in natural language processing (NLP) and plays a key role in structuring text efficiently [1,2]. The recent advances in generative large language models (LLMs) have proven highly beneficial across various IE tasks in several domains [3]. Within the health care sector, both researchers and clinicians are actively seeking to extract valuable insights from unstructured clinical documents [4]. Leveraging LLMs for addressing IE tasks in this specific domain promises to expedite and enhance clinical operations, thereby improving clinical decision-making and documentation quality, which results in better patient care [5,6].

Named entity recognition and named entity normalization (NER and NEN) serve as crucial IE tasks within the medical domain [7]. NER focuses on accurately identifying entities of interest, such as findings, diseases, medications, and procedures. Subsequently, NEN performs the standardization of these entities using referencing standards such as concept ontology or knowledge bases. These tasks play a fundamental role in various health care applications, facilitating interoperability between different medical systems.

State-of-the-art approaches often frame the NER task as a sequence labeling problem, which is well-suited for continuous named entities (NEs). However, NE words may sometimes appear nonconsecutively, forming what is known as Discontinuous Named Entities (DiscNEs) [8]. These entities may also be referred to as “disjoint spans” or “disjoint mentions.” Conventional sequence labeling schemes are insufficient to handle DiscNEs, and specialized schemes, although tailored, often lack generalizability [9]. Therefore, this study aims to explore the effectiveness of generative LLM-based models as a unified solution for identifying both continuous and discontinuous NEs within a newly introduced benchmark dataset, as part of the BioCreative VIII shared task [10,11].

Until recently, the ongoing series of BioCreative challenges contributed significantly to advancing the clinical NLP community by providing benchmark datasets to evaluate the effectiveness of IE models. The eighth edition of the BioCreative challenge, organized into 4 tracks, includes our participation in the third track. This track focuses on the extraction and normalization of genetic phenotypes in clinical reports [10]. As part of this track, participants were provided with patient physical examination reports comprising both normal and key findings. Normal findings (NORMFs) typically denote the absence of phenotypic abnormalities in the observation (eg, “intact palate”), while key findings (KEYFs) indicate the presence of phenotypic abnormalities (eg, “high-arched palate”). The tasks involve extracting KEYFs while excluding NORMFs, as they are not represented in the human phenotype ontology (HPO), and then mapping these KEYFs to their corresponding HPO identifiers [12].

**Figure 1:**
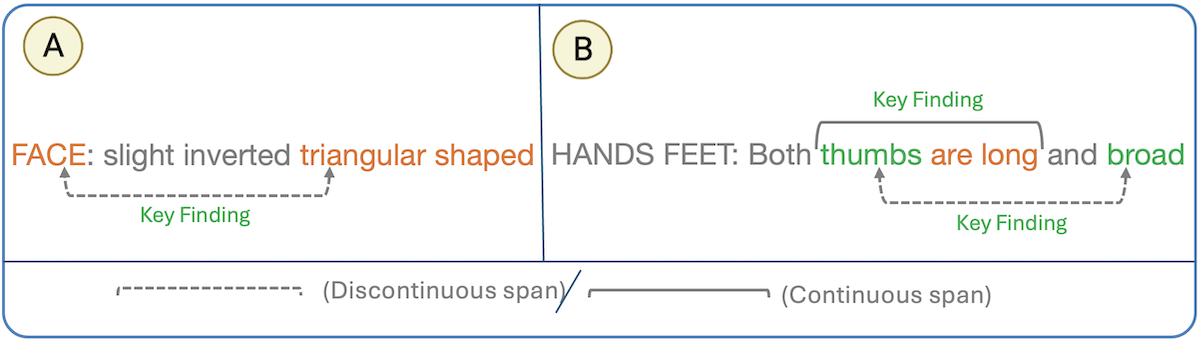
Examples of discontinuous named entities: (A) nonoverlapping and (B) overlapping disjoint mentions.

A significant challenge revolves around the presence of DiscNEs in the text. Approximately 14% of mentions in the training and validation sets can be considered as discontinuous (465 DiscNEs out of 3247 NEs). We observed 2 forms of DiscNEs in the dataset: either one entity is interrupted by nonentity tokens (nonoverlapping disjoint mentions), or multiple entities share a common head, with one or more entities interrupting the other (overlapping disjoint spans). Figure 1 demonstrates examples of the 2 forms of discontinuity found in the clinical entries, where example (A) presents a nonoverlapping case. The gold standard annotations mark the span “FACE triangular shaped,” as a KEYF. As we can see, the nonentity tokens, “slightly inverted,” interrupt the original span. This example is considered to be nonoverlapping as none of its tokens are shared with another mention. As for Example B, an instance of overlapping disjoint spans is demonstrated. Here, the token “thumbs” acts as a shared head between the 2 KEYFs, “thumbs are long” and “thumbs are broad.” The task organizers encouraged addressing this challenge, urging solutions that go beyond traditional sequence labeling methods, effective only in identifying entity spans with consecutive tokens, and falling short in extracting DiscNEs [10].

To accomplish this task, we introduce DiscHPO, a 2-component pipeline. The first component involves a sequence-to-sequence approach for detecting continuous and DiscNE spans, while the second component focuses on normalizing the extracted spans to their associated HPO identifiers using the Sentence Transformers framework. The DiscHPO pipeline can enhance downstream tasks such as diagnostic decision support. For instance, integrating our system into electronic health record (EHR) platforms could enable real-time identification of phenotypic patterns relevant to rare diseases, potentially accelerating diagnosis. Furthermore, normalization to standardized vocabularies like HPO facilitates interoperability and large-scale phenotypic analyses, ultimately supporting more personalized and data-driven health care delivery.

### The HPO

The HPO was introduced in 2008 as a comprehensive logical framework intended to describe and analyze phenotypic abnormalities in patients with a particular disease [12]. It offers a standardized representation of phenotype concepts. The use of HPO not only enhances clinical care and education but also serves as a valuable resource for EHR analysis and structuring, facilitating accurate diagnosis of diseases. Thus, the automatic extraction of a thorough and precise set of HPO terms directly from the EHR data is highly desirable to reduce the burden of manual annotation and save time and resources [13].

In this study, we use a version of the HPO dictionary provided by the shared task organizers. This version encompasses around 17,000 terms, which include both the preferred term for the phenotypic abnormality and the corresponding HPO identifier. In addition to this, the organizers provided a supplementary list of 5000 terms that were not observed in the corpus, which has been helpful in refining the original list of terms.

### Prior Work

We first explore the existing work in biomedical NER and normalization. Conventionally, NER is followed by an NEN step (2-stage pipeline), where identified mentions are mapped to standardized concepts within controlled vocabularies. For instance, Peng et al [14] introduced a standalone normalizer that receives input mentions from an external NER component. Their method uses an interaction-based synonym marginalization technique that effectively captures the relationships between a given NE and its candidate matches. Similarly, Ji et al [15] proposed a BERT (bidirectional encoder representation from transformers)-based ranking method for biomedical entity normalization, showing the power of contextualized embeddings in disambiguating similar terms.

Beyond these 2-stage methods, several studies have explored joint or end-to-end frameworks that integrate NER and normalization tasks to reduce error propagation and exploit shared contextual representations. Xiong et al [16] proposed a joint model that treats NER as a machine reading comprehension task and NEN as a multilabel sequence tagging problem. Zhao et al [17] further advanced this line of work with a multitask learning framework that jointly models both NER and normalization, incorporating explicit feedback strategies to enhance performance.

We now review studies dedicated to NER and normalization to the HPO. Increasing attention has been given to the automated identification of HPO concepts from clinical narratives. For example, Son et al [18] introduced EHR-Phenolyzer to extract phenotypes from EHR narratives. Their approach involves the use of 2 clinical IE tools, namely MedLEE [19] and MetaMap [20]. They found that both systems performed comparably to expert annotation. Since the earlier approach relies on general NLP tools, a more tailored phenotypes extraction approach, ClinPhen is introduced by Deisseroth et al [21], which uses the natural language toolkit and a hash table for the extraction and normalization. ClinPhen showed superior performance compared with general IE tools: MetaMap and cTAKES [22]. A comparative evaluation study conducted by Liu et al [23] included comparing the following HPO extraction models: MetaMapLite, MedLEE, ClinPhen, cTAKES, as well as 4 ensemble techniques. Their findings show that ensemble methods consistently surpass individual models in recognizing both generic and patient-specific phenotypic concepts.

Other approaches have developed interactive platforms for extracting HPO terms. For instance, Doc2HPO [24] provides a web interface for real-time extraction and normalization of HPO terms. Another platform, Monarch Initiative [25], serves as a comprehensive platform for integrating phenotypes and genotypes data and analytics. However, these dictionary-based approaches (MetaMapLite, cTAKES, ClinPhen, and Doc2HPO) use dictionary matching as their primary method, depending exclusively on exact matches with dictionary terms. This approach proves challenging in capturing semantic and syntactic variations within terms [26].

Alternatively, machine-learning approaches have shown efficacy in understanding textual semantics. For instance, the PhenoTagger system [26] is a hybrid approach merging dictionary-based matching and machine-learning classification to identify HPO concepts. Inspired by PhenoTagger, Feng et al [27] introduced PhenoBERT. While both methods use BERT [28] as their core model, PhenoBERT distinguishes itself by incorporating 2-level hierarchical CNNs. This integration serves to narrow down the BERT search space, which results in a reduction in computational time.

For identifying overlapping mentions, PhenoTagger has incorporated several heuristic rules to handle nested mentions and those sharing common tokens. Additionally, PhenoBERT has introduced a phrase recovery step to handle simple coordination ellipses. However, both models still encounter difficulties in resolving overlapping DiscNEs with gaps longer than one token, as in the example in Figure 1B. Moreover, they struggle to recognize nonoverlapping DiscNEs (Figure 1A).

### Goal of This Study

Although sequence labeling and deep learning methods have been widely used to recognize both continuous and discontinuous NEs, our study aims to incorporate a generative model as the NER component within our DiscHPO pipeline. We explore the capabilities of these models in detecting both continuous and discontinuous genetic phenotype mentions in physical examination reports. Our goal is to deliver an integrated solution that recognizes and normalizes these mentions within a consistent framework.

The main contributions of our work are as follows:

1. An investigation into the effectiveness of open-source generative pretrained language models (PLMs) for extracting both continuous and discontinuous spans.
2. The integration of a Sentence-Transformers model with the NER component to effectively normalize the extracted mentions to the HPO.

## Methods

### Dataset

The BioCreative VIII shared task dataset [11] comprises deidentified reports documenting clinical observations from dysmorphology physical examinations. It consists of 3136 observations, distributed among a training set (1716 observations), a validation set (n=454), and a test set (966 + 2427 decoy observations). Each observation may encompass one or more entity spans categorized as either key or NORMF entities.

Table 1 displays a snippet featuring 2 entries from the provided gold standard annotations. The dataset includes 5 columns: an observation ID assigning a unique identifier to each observation, raw observation text, an HPO column containing an HPO ontology identifier associated with the mention, a Polarity column indicating “X” for NORMFs and “NA” for KEYFs, and a Spans column encompassing the character offsets of the entity mention.

**Table 1:** Example snippet of the gold standard annotations from the training set.

Observation ID	Text	HPO^a^	Polarity	Spans
F8D319161DE176F2	FACE: No facial abnormalities noted within the limitations of examination.	HP:0000271	X^b^	6-35
DC00B8A9EDA2C78	HANDS FEET: Both thumbs are long and broad	HP:0011304	N/A^c^	17-27 and 37-42
DC00B8A9EDA2C78	HANDS FEET: Both thumbs are long and broad	HP:0032524	N/A	17-32

^a^HPO: human phenotype ontology.

^b^Normal findings

^c^N/A: not applicable.

As shown in Table 1, discontinuous spans are presented with 2 offset ranges separated by a comma. In the case of overlapping spans, each span is represented in a separate row with its assigned HPO identifier, ensuring the preservation of all HPOs associated with the observation. Furthermore, there are 2 levels of segmentation, where disjoint mentions can be divided into 2 or 3 constituent parts. Table 2 provides a numerical analysis of the corpus.

**Table 2:** Analysis of the BioCreative VIII task 3 corpus, including the training and validation sets.

Analysis	Training set	Validation set
Number sentences, n	1716	454
Number entities, n	2562	685
Number disjoint mentions, n	369	96
Percent of disjoint mentions, %	14.4	14
Disjoint (2-constituent parts), n	358	91
Disjoint (3-constituent parts), n	11	5
Normal findings, n	329	78
Key findings, n	2233	607

During the competition evaluation period, the test set was provided, comprising solely observation IDs and raw text entries. It consists of 966 deidentified observations, along with 2427 decoy observations. However, the gold-standard annotations were not provided to participants to ensure a level playing field.

In addition to this dataset, we aimed to assess the generalizability of our model under different conditions using the GCS+ dataset [29], a widely adopted benchmark that has been used by several models for HPO term extraction and normalization. The dataset contains annotated HPO terms along with their corresponding HPO IDs. Its structure often includes nested named entities, but DiscNEs are not explicitly annotated, despite their presence in the text. We followed the data splits provided by Yang et al [30] and further fine-tuned our DiscHPO checkpoint on the GCS+ dataset.

### Data Preprocessing

We approach the NER task by framing it as a sequence-to-sequence NER problem [31]. In this setup, the system takes a sequence of tokens as input and generates a target sequence containing recognized spans categorized as either NORMF, KEYF, or not applicable entities. Unlike sequence labeling, where models assign tags to individual tokens, sequence-to-sequence models learn to produce a new sequence of tokens that transforms the input sequence. To align with this approach, preprocessing of NER system inputs and targets is necessary before model training. Both datasets underwent the same preprocessing steps.

First, we convert the numerical offsets of entity spans into word spans. Each span is then prefixed with its corresponding entity type. Subsequently, all entity spans related to a single observation are consolidated into one sequence, separated by a semicolon. Both the training and validation sets undergo the same preprocessing steps. An example of a preprocessed target is illustrated in Table 3. By using this scheme, DiscNEs can be represented more effectively since no special labels are used to distinguish the disjoint entities. After preprocessing the BioCreative dataset, we reviewed the dataset and identified minor inconsistencies in the textual spans, primarily caused by inaccurate numerical offsets. We manually corrected these issues and provided the complete list of revised entity spans in Table S1 in Multimedia Appendix 1.

**Table 3:** Preprocessed named entity recognition inputs.

Input	Target
EYES: Sparse eyebrow hair. Normal lids and lashes.	KEYF^a^: Sparse eyebrow; NORMF^b^: Normal lashes; NORMF: Normal lids
NECK: Excess nuchal skin noted with no webbing, cysts.	KEYF: Excess nuchal skin; NORMF: NECK: no webbing; NORMF: NECK: no cysts

^a^KEYF: key finding.

^b^NORMF: normal finding.

For the training phase, the model input consists of the context, including the input sentence, followed by the corresponding predictions, which include all textual named entities and their types. During inference, the model is given just the sentence and is tasked with generating the NE predictions.

After the model predicts the spans, a postprocessing step is performed to convert the spans back into numerical offsets by locating the offset positions of a given span within the observation text using regular expressions. This step is essential to ensure compatibility with the format specified in the evaluation script provided by the task organizers when calculating the validation set prediction results.

### DiscHPO Pipeline

**Figure 2:**
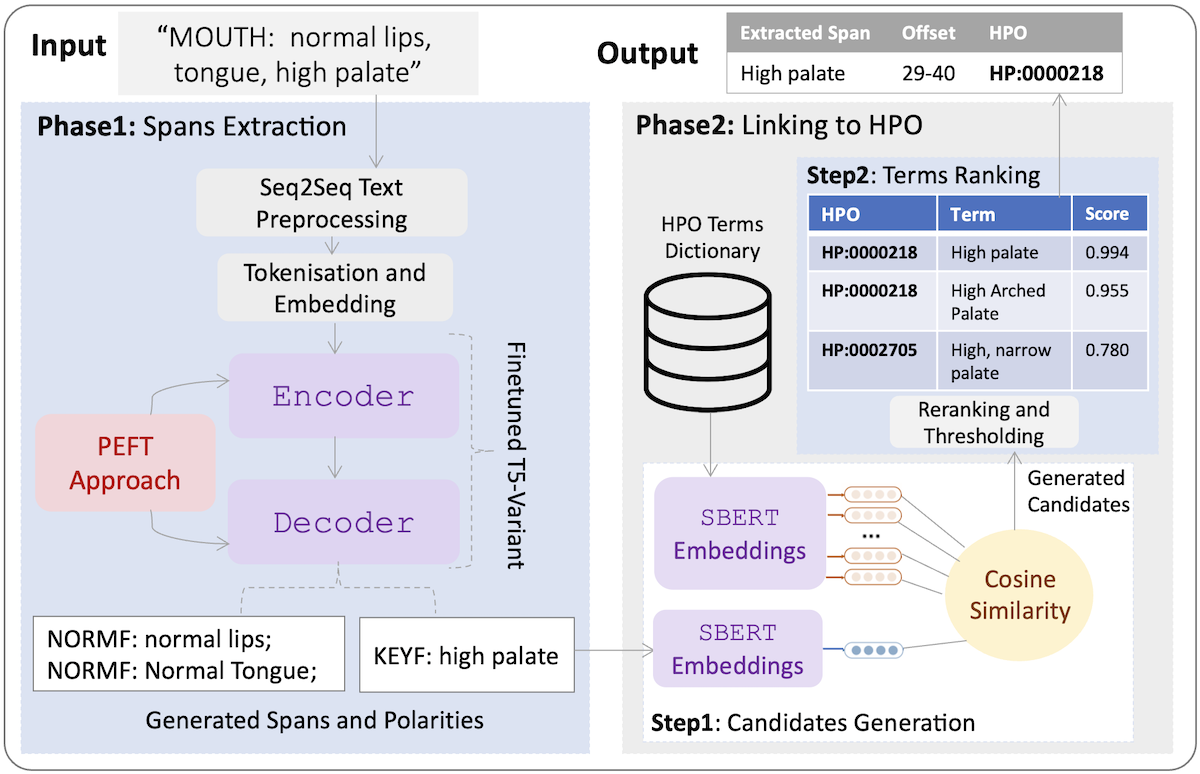
DiscHPO pipeline architecture. HPO: human phenotype ontology.

We designed DiscHPO as a pipeline of 2 components: a sequence-to-sequence NER model that can recognize both continuous and discontinuous mentions at the sentence level, and an entity normalizer underpinned by a sentence transformer bidirectional encoder (biencoder) for candidate generation and a cross-encoder reranker. The DiscHPO pipeline is illustrated in Figure 2.

### The NER Model

We implemented a sentence-level NER system by fine-tuning a pretrained sequence-to-sequence encoder-decoder language model based on the transformers architecture [32]. Our approach involved leveraging different versions of the text-to-text transfer transformer (T5) [33]. The T5 model adopts a unified approach to transfer learning, transforming various text processing tasks (eg, question answering, language modeling, or span extraction) into a “text-to-text” problem. The model underwent pretraining through a consecutive span denoising objective and uses the standard maximum likelihood training method, incorporating teacher forcing. The generative capability of T5 enables seamless fine-tuning for the extraction of the span and prediction of their target class simultaneously. Consequently, the NER component will be capable of distinguishing both types of mentions. (ie, normal and KEYFs).

Each observation and its corresponding spans undergo tokenization using the T5 tokenizer, which is built upon SentencePiece [34]. The tokenizer breaks down the sequences into tokens, translates these tokens into IDs, pads them to achieve the maximum sequence length, and appends the end-of-sequence token (</s>). As we are not engaging in multitask training, there is no need for a task prefix at the beginning of the text (eg, “NER:”).

For the model fine-tuning, we examined the following T5 architectures:

- The original T5 [33], which is based on the transformers’ encoder-decoder architecture and pretrained with the Colossal Clean Crawled Corpus.
- FLAN-T5 [35], or (Fine-tuned LAnguage Net-T5), which is an improved version of T5, instruction-finetuned across a variety of tasks, including NER.

We also considered experimenting with a domain-specific (ie, biomedical) variant of T5, which is the following:

- SciFive [36], which differs by pre-training a T5-architecture model on biomedical articles from the PMC and abstracts from PubMed.

Table S2 in Multimedia Appendix 1 outlines the optimal hyperparameter settings used in all T5 architectures throughout our NER experiments.

To optimize the NER model, a parameter-efficient fine-tuning method such as low-rank adaptation (LoRA) [37] is used, eliminating the hardware costs associated with fine-tuning LLMs by using a small portion of the pretrained parameters (around 1.1%). To do this, LoRA reparametrize (or decomposes) the required weight updates (ΔW) based on the original pretrained weight matrix W_0_ ∈ ℝd×k, where d and k are the dimensions of the weight matrix. The low-ranked decomposed matrix is the result of the multiplication of LoRa matrices A and B:

∆W = BA (1)

where B ∈ ℝd×r, A ∈ ℝr×k, and the rank r ≪ min(d,k).

Hence, the sizes of the A and B matrices are adjusted using LoRa rank (r). However, for the initial scaling of the update weights, 2 hyperparameters of LoRa: the rank (r) and a constant alpha (α) are used as in the equation:

∆W= α / r (2)

We used the Python package loralib, to implement LoRa. Specifically, we chose the extra-large version of Flan-T5 (Flan-T5-XL). After exploring various combinations of r and α values, we achieved optimal results when setting r to 16, and α set to values of both 512 and 1024.

### Normalization to HPO

**Figure 3:**
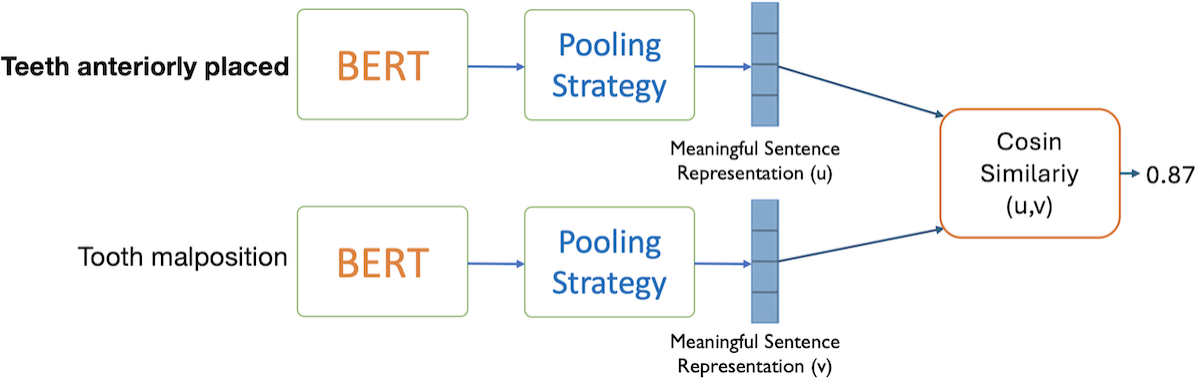
SBERT general Siamese network architecture. SBERT: sentence bidirectional encoder representation from transformers.

The second task of this challenge involves normalizing the identified KEYFs to their HPO identifiers. We use Sentence-transformers (or SBERT [sentence bidirectional encoder representation from transformers]), a Python framework, that excels in embedding sentences, text, and images, demonstrating state-of-the-art performance in sentence-pair regression tasks [38,39]. Figure 3 illustrates the main design aspects of SBERT. Specifically, it is a modified version of BERT that uses Siamese networks to generate sentence embeddings with semantic similarity. These embeddings can be compared efficiently using cosine similarity, resulting in a significant reduction in computational overhead [40]. A crucial aspect of SBERT’s design involves the pooling operation, used to aggregate the output embeddings of BERT into a fixed-sized sentence embedding. Commonly used pooling strategies include (1) using the (CLS) token embedding of BERT, (2) mean pooling (averaging all token embeddings), and (3) max pooling (taking the maximum value across embeddings). In our experiments, we opted for mean pooling as it offers a favorable balance between computational efficiency and representational effectiveness, being less resource-intensive compared with other aggregation strategies. For a detailed comparative analysis of these pooling methods and their impact on performance, we refer readers to the study by Xing et al [41].

In our study, we leverage this framework to conduct a semantic textual similarity task between pairs of extracted mentions and HPO concepts sourced from the provided dictionary. This framework involves two steps: (1) candidate generation and (2) reranking.

The candidate generation step includes identifying candidate terms from the HPO dictionary. In this step, the mention and each candidate HPO term are jointly encoded to compute a semantic similarity score, which reflects how closely related the 2 texts are in meaning. This score typically ranges from 0 to 1, where 1 indicates perfect semantic equivalence and 0 denotes complete dissimilarity. The higher the score, the more semantically aligned the candidate is with the input mention.

**Figure 4:**
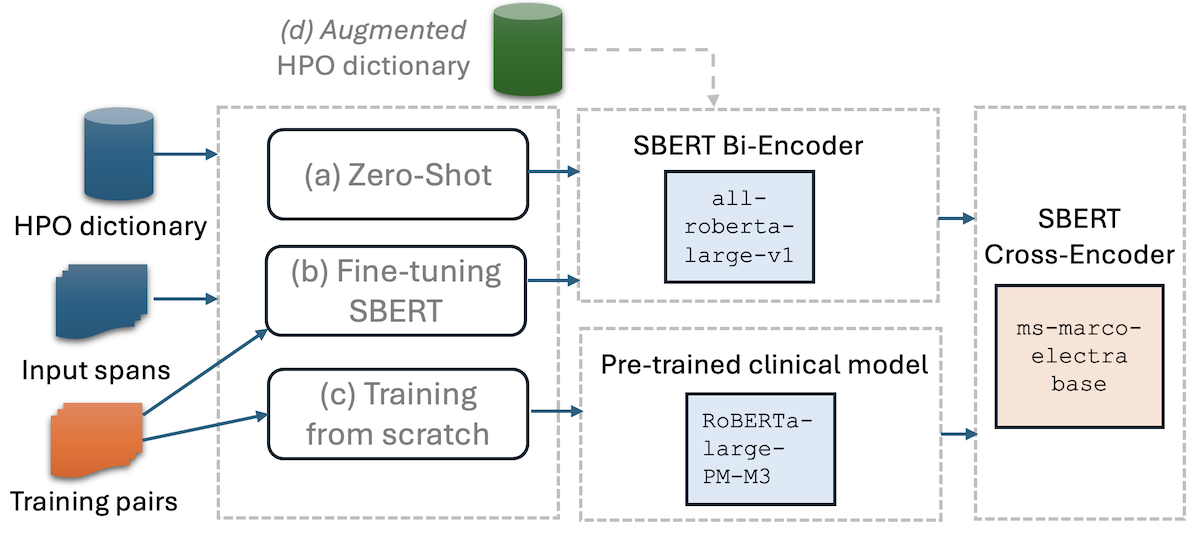
Preliminary exploration of different settings for 3 training paradigms of Sentence Transformers. Configurations in (A) and (B) employ an existing SBERT biencoder, whereas (C) involves training a non-SBERT pretrained language model from the clinical domain. In (D), we investigate a setting where an augmented HPO dictionary with synonyms is used for both (A) and (B). HPO: human phenotype ontology; SBERT: sentence bidirectional encoder representation from transformers.

Using the 734 gold standard spans from the validation set, we conducted a preliminary investigation using different sentence transformer biencoders and exploring 3 training paradigms within the framework (Figure 4). These paradigms include (1) using an existing sentence transformer biencoder without training (zero-shot setting), (2) continuing training (fine-tuning) the Sentence Transformer biencoder, or (3) training a non-Sentence Transformer PLM from scratch by defining the SBERT individual layers.

For the latter 2 scenarios, we used pairs of spans from the training set and their corresponding HPO terms to fine-tune SBERT-biencoder and train a clinical PLM from scratch on a semantic similarity objective. We used MegaBatchMarginLoss as the training loss function, which is tailored to our training paradigm involving 2 pairs: an anchor and a positive example demonstrating semantic similarity to the anchor. Moreover, as illustrated in Figure 4D, we sought to enhance the generated candidates’ accuracy by using an augmented HPO dictionary with synonymous terms sourced from the training dataset.

Based on the insights gained from this investigation, we select the optimal setting for our DiscHPO normalization component. We found that fine-tuning an existing SBERT biencoder (all-roberta-large-v1) with an augmented HPO dictionary, which incorporated synonyms from the training set, proved to be the most effective approach to create embeddings for both HPO terms and the extracted entity spans.

The reranking step is when the candidate results are reranked using a cross-encoder, which reorders the candidates using their similarity score, ensuring that the most semantically relevant HPO concepts are prioritized at the top of the list. Specifically, the top 30 candidates obtained by semantic matching are then passed on to a sentence transformer cross-encoder model. After experimenting with several cross-encoders, we found that ms-marco-electra-base yielded the highest results. It reranks the generated candidates by calculating the scores for each span-candidate term combination. Its results are sorted to produce the final reordered HPO terms. Among these results, only the top score is considered for submission.

### Evaluation Metrics

For evaluation, we adhered to the official evaluation script provided by the task organizers. It incorporates the Precision (P), Recall (R), and *F*_1_-score (*F*_1_) metrics. The equations for these 3 metrics are as follows:

Precision = True Positive / (True Positive + False Positive)

Recall = True Positive / (True Positive+False Negative)

*F*_1_-score = (Precision * Recall) / (Precision + Recall)

There are 2 evaluation scenarios implemented in the provided evaluation script:

- Evaluation of normalization task: which is calculated solely from the normalized output (the HPO term ID) of the extracted span. Here, the span extraction result is not part of the calculation.
- Evaluation of a combined span extraction and normalization: which measures the performance of both steps taken together.

To evaluate the latter, metrics for span extraction were reported based on exact matching (where the character offsets of the KEYF must be identical with the gold standard annotations) and partial matching (which considered an extracted span to be correct if it overlaps with the gold standard span with at least one correct token). In all evaluation experiments, we maintain consistent settings for the normalization component while experimenting with different NER models that are based on T5.

### Baselines

To assess the effectiveness of our proposed approach, we benchmark it against 2 baseline models:

- PhenoTagger [26]: A hybrid approach that combines an HPO dictionary with machine learning techniques, leveraging several PLMs such as Bioformer, BioBERT, and PubMedBERT. In our work, we adopt the evaluation by Weissenbacher et al [42] where BioBERT served as the base model.
- PhenoID [42]: This system follows a 2-phase architecture, comprising an extractor and a normalizer. The extractor is a sequence labeling approach that leverages a transformer-based model, specifically the Bio-ClinicalBERT. For normalization, the task is treated as a classification problem by fine-tuning Bio-ClinicalBERT.

For the GCS+ dataset, we benchmarked our model against PhenoTagger and 2 SOTA models:

- PhenoRerank [43]: This model builds on a BlueBERT backbone pretrained on a weakly supervised dataset that includes HPO concepts, synonyms, definitions, and comments from the HPO database. The model is trained using a contrastive loss and reranks its predictions using external HPO extraction tools.
- PhenoGPT [30]: Based on GPT-3 and pretrained on a dataset derived from EHRs, literature, and phenotypic databases. The final PhenoGPT model is further fine-tuned on the GCS+ dataset to optimize its performance.

### Ethical Considerations

This study makes use of the BioCreative VIII shared task dataset, which is publicly available. The original shared task paper provides details on the data collection process and sources [11]. As this work does not involve any primary data collection or experimentation with human participants, no ethics approval is required.

## Results

We start by presenting our DiscHPO validation results: the extracted findings and their normalized HPO IDs (Table 4).

**Table 4:** DiscHPO results on the validation set.

Model	Normalization				Exact extraction and normalization				Partial extraction and normalization			
	Precision	Recall	*F*_1_-score	Precision		Recall	*F*_1_-score	Precision		Recall	*F*_1_-score	
SciFive-Large	0.722	0.726	0.724	0.695		0.637	0.665	0.719		0.719	0.719	
T5-Large	0.725	0.743	0.734	0.696		0.644	0.669	0.724		0.739	0.732	
FlanT5-Large	0.708	0.731	0.719	0.678		0.637	0.658	0.708		0.729	0.718	
FlanT5-XL LoRa 512	0.735	0.739	0.737	0.710		0.654	0.681^a^	0.734		0.738	0.736	
FlanT5-XL LoRa 1024	0.743	0.739	0.742^a^	0.716		0.642	0.677	0.742		0.735	0.738^a^	

^a^Best-performing values.

Since FlanT5-XL showed the best performance in the evaluation stage, in the testing phase, we submitted 3 runs based on this model. Same as the evaluation, we applied the same normalization component in all runs while using different NER settings:

- Run1: FlanT5-XL and LoRa, α=1024 trained on the training set only.
- Run2: FlanT5-XL and LoRa, α=1024 trained on both the training and validation sets.
- Run3: FlanT5-XL and LoRa with α=512 trained on the training set only.

As shown in Table 5, the best normalization score of 0.723 *F*_1_-score is achieved by using FlanT5-XL with LoRa α of 512.

**Table 5:** DiscHPO results on the test set submitted to the competition.

Runs	Normalization				Exact extraction and normalization				Partial extraction and normalization		
	Precision	Recall	*F*_1_-score	Precision		Recall	*F*_1_-score	Precision		Recall	*F*_1_-score
1	0.721	0.723	0.722	0.694		0.638	0.665^a^	0.72		0.721	0.72
2	0.707	0.731	0.719	0.674		0.625	0.648	0.706		0.727	0.716
3	0.718	0.728	0.723^a^	0.69		0.637	0.662	0.717		0.726	0.721^a^

^a^Best-performing values.

The test set results are comparable to the validation set, which indicates that the model’s performance is consistent across different sets. Overall, the highest normalization improvement was obtained by using FlanT5-XL with LoRa α=512.

In Table 6, we compare our highest-performing submission with the baselines on the same dataset, demonstrating the superiority of DiscHPO in terms of Recall and *F*_1_-score. We also compare with the best-performing participants in the shared task. PheNormGPT [44] achieves the best performance by leveraging few-shot learning with GPT-4. This model generates 2 outputs for each span: the extracted span from the text and a preferred term generated by GPT-4. While the results are robust, this model has not been validated in a multiclass setting, where only KEYFs were extracted. In addition, although the BioCreative dataset is deidentified, the nonlocal deployment of GPT-4 raises privacy concerns when used with nondeidentified clinical data due to the potential disclosure of protected health information [45]. In contrast, open-source models such as the T5 family offer full local deployment on authorized machines, ensuring better control over data privacy, while also providing a more cost-effective alternative to proprietary models like GPT-4. W2NER+PhenoTagger [46] comes in second place. The model uses the W2NER framework for NER [47], which formulates NER as a grid-tagging problem. It uses a grid structure to capture all word relationships. However, this approach depends on a predefined tagging scheme. In contrast, our model does not rely on custom tagging structures, offering flexibility in handling diverse entity types.

**Table 6:** Best DiscHPO results on test set, compared against baseline methods [1,5] and the top 2 performing models from the BioCreative VIII Track 3 shared task.

System	Normalization				Partial extraction and normalization		
	Precision	Recall	*F*_1_-score	Precision		Recall	*F*_1_-score
PhenoTagger	0.587	0.687	0.633	0.586		0.685	0.632
PhenoID	0.736	0.699	0.717	0.735		0.696	0.715
W2NER+PhenoTagger	0.831	0.706	0.763	0.830		0.704	0.762
PheNormGPT	0.842	0.799	0.820^a^	0.841		0.794	0.817^a^
DiscHPO (Ours)	0.718	0.728	0.723	0.717		0.726	0.721

^a^Best-performing values.

To assess model performance in differentiating between normal and KEYFs, we calculated class-wise *F*_1_-scores. The model demonstrates a stronger ability to recognize KEYFs (77.2% *F*_1_-score; 471 true positives, 142 false positives, and 135 false negatives) compared with NORMFs (56.4% *F*_1_-score; 44 true positives, 34 false positives, and 34 false negatives), reflecting a recognition bias. This bias is expected and can be attributed to the notable class imbalance in the dataset, where NORMFs constitute only 12% of the total entity spans across the combined training and validation sets (Table 2). Given that the primary goal of the shared task is the detection and normalization of KEYFs, where NORMFs are excluded from the final evaluation, this class imbalance has minimal impact on overall task performance. Consequently, our model was optimized to prioritize KEYFs, as they are the only entities passed to the normalization phase.

To evaluate the models’ proficiency in specifically identifying disjoint spans, we computed the NER results for the top 4 DiscHPO models by incorporating only the extracted disjoint mentions from the validation set. We computed both the exact and partial extraction scores for each model. As shown in Table 7, our NER models can identify discontinuous spans. As can be seen, FlanT5-XL with LoRa α 512 is superior in detecting disjoint mentions of findings with an *F*_1_-score of 63.1%. In terms of overlapping extraction, FlanT5-XL with LoRa α=1024 has the best performance when it comes to extracting some of the parts of the disjoint mentions.

**Table 7:** Results for discontinuous span extraction on the validation set.

Model	Exact extraction				Partial extraction		
	Precision	Recall	*F*_1_-score	Precision		Recall	*F*_1_-score
T5-Large	0.546	0.575	0.57	0.873		0.937	0.904
SciFive-Large	0.6	0.578	0.589	0.911		0.868	0.889
FlanT5 LoRa 512	0.699	0.569	0.631	0.79		0.823	0.806
FlanT5 LoRa 1024	0.658	0.578	0.615	0.927		0.881	0.904

In Table 8, we further analyzed the model’s behavior across structural variations in discontinuous entities, including spans without interrupting tokens and those with shared heads (ie, overlapping tokens) with varying head locations and structural complexity. The *F*_1_-score was highest for left-overlapping spans and spans with no overlap. Right and multiple overlaps also showed reasonable performance, with some variation that correlates with the number of instances in each category (eg, 19 and 12 spans respectively, compared with 40 for left overlaps).

**Table 8:** Model’s performance across different discontinuous structures.

DiscNE^a^ category	Precision	Recall	*F*_1_-score
No overlap	0.69	0.67	0.68
Left overlap	0.71	0.62	0.67
Right overlap	0.50	0.46	0.48
Multiple overlaps	0.83	0.45	0.59

^a^DiscNE: discontinuous named entity.

To assess the generalizability of the model, we show the performance results of DiscHPO on the GCS+ dataset in Table S3 in Multimedia Appendix 1. We have also included a discussion of these results in comparison with the baseline models.

## Discussion

### Principal Findings

It is noteworthy that, in both our system and the compared ones, the scores for partial extraction and normalization are very close to those of normalization, which suggests that normalization performance is not substantially degraded by partially extracted spans. This implies that exact span extraction is not always a condition for accurate normalization. This can be observed in cases where partially extracted spans did not negatively affect the normalization outcome.

In around 15 normalization output examples from the validation set, we found that partial extraction of continuous and discontinuous NEs can be sufficient for normalization. This is especially true when a disjoint span is interrupted by nonentity tokens and does not share a head. If we consider the following example: “EYES: normal brows, mild hooding.” While all our models accurately extracted the exact disjoint span “EYES: hooding,” we further examined this by inputting the normalizer with the partial span “hooding.” This also resulted in correct normalization, as the term “hooding” is commonly associated with an eye condition.

### Quantitative Error Analysis

**Figure 5:**
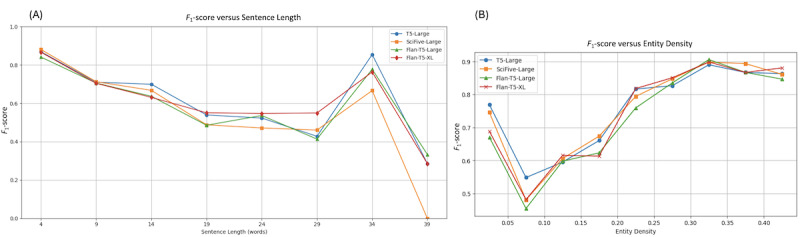
Quantitative error analysis of DiscHPO. It compares the F1-score performance of 4 backbone pretrained language models (T5-Large, SciFive-Large, Flan-T5-Large, and Flan-T5-XL) under two factors: (A) the impact of sentence length and (B) the impact of entity density. HPO: human phenotype ontology.

Figure 5 presents a comparative analysis of the *F*_1_-scores achieved by T5-Large, SciFive-Large, Flan-T5-Large, and Flan-T5-XL-LoRa1024, which we used as a backbone PLMs for DiscHPO, across different sentence lengths (Figure 5A) and varying levels of entity density (Figure 5B).

We first examine the impact of sentence length on *F*_1_-score. In Figure 5A, we observe a general decline in *F*_1_-score as sentence length increases across all 4 models. This trend is expected, as longer sentences contain more complex syntactic structures. However, T5-Large shows increased performance, particularly in longer sentence ranges, suggesting robustness to increased sequence complexity. SciFive-Large and Flan-T5-Large show relatively parallel performance, though SciFive-Large shows a sharper drop at the longest sentence bin. Flan-T5-XL demonstrates a stable performance across medium-length sentences. We can observe a spike in performance in the 34-word bin for all models, suggesting a concentration of entity-rich sentences in that length category.

Next, we explore the impact of entity density, defined as the percentage of annotated NEs within a sentence. Figure 5B shows that model performance improves with increasing NE density, peaking around the 0.3-0.35 density range. This trend indicates that models benefit from contexts rich in NEs, which may provide clearer clues for learning entity boundaries and relationships. Flan-T5-XL achieves the highest performance at higher density levels, suggesting strong capabilities in recognizing entities in dense annotations, due to its larger parameter capacity and better instruction tuning. Other models perform comparably with each other, but Flan-T5-Large performs slightly lower, suggesting potential limitations in handling low-entity sentences.

### Qualitative Error Analysis

From our analysis of the gold DiscNEs, we found that certain tokens are deemed nonessential for normalization. For example, in the instance “EYES: Mild epicanthus, mild up-slant,” the gold standard span “EYES - mild up-slant” is disjoint. However, none of our models incorporated the severity level “mild” into the extracted span; this omission did not adversely impact the normalization outcome.

On the other hand, in certain examples, specific tokens in disjoint mentions are crucial for achieving exact normalization. For example, in the case of “HANDS FEET: L clinodactyly,” the gold annotation is the disjoint span “hand L clinodactyly,” whereas all our models extracted only “clinodactyly.” This led to normalization as “clinodactyly” encompassing both fingers and toes, instead of the more specific concept “Finger clinodactyly.”

The case is different when disjoint spans share a common head, in which each span must be resolved to its own head and its constituent tokens to avoid missing normalizations. We also observed that the models easily identified simple coordinated ellipses, such as “NOSE: broad nasal bridge and tip.” However, challenges arise in scenarios where 2 spans share 2 heads, as in the example “Deep palmar and plantar creases,” where the tokens “deep” and “creases” are common to both “palmar” and “plantar.” Scifive struggled with this complexity, failing to accurately resolve the second span and consequently missing its normalization. In contrast, the other 3 models effectively handled this complexity and successfully addressed the shared elements.

Shifting to normalization errors, some can be attributed to the lack of descriptions or synonyms for the HPO concepts. These additional details provide the normalizer with additional information for accurate concept identification. For instance, in the first example from Table 9, the extracted span “white plaque under the tongue” led the normalizer to mistakenly assign the concept “Erythematous plaque” instead of “Oral leukoplakia” due to lexical similarity. This type of error occurred 28 times out of 155 total normalization errors in the validation set. While we have already enhanced the normalizer’s training data with synonyms from the original training set, they might be inadequate for unseen examples in the validation set. Therefore, we assume that adding more HPO synonyms or descriptions may have the potential to improve the normalization process.

**Table 9:** Examples of human phenotype ontology (HPO) terms incorrectly predicted by the normalizer on the validation set.

Gold and predicted span	Gold Standard HPO			Predicted HPO	
	Concept	HPO ID	Concept		HPO ID
White plaque under the tongue	Oral leukoplakia	HP:0002745	Erythematous plaque		HP:0025474
Hypotonia	Hypotonia	HP:0001252	Generalized hypotonia		HP:0001290
Abdominal wall hernia	Hernia	HP:0100790	Hernia of the abdominal wall		HP:0004299
Flat-nasal bridge	Depressed nasal ridge	HP:0000457	Depressed nasal bridge		HP:0005280

Another example of error arises when our model selects the term “Generalized hypotonia,” instead of the more specific term “Hypotonia” as provided by the gold standard. Conversely, another error arises when our model selects the highly precise concept “Hernia of the abdominal wall,” while the gold standard prefers the broader term “Hernia.” This type of error was observed in 5 instances among all normalization errors. In addition, we observed only 8 inconsistencies among the gold standard annotations, as illustrated in the final example of Table 9. In this case, the term “Depressed nasal bridge” was mistakenly recorded as “Depressed nasal ridge,” and has been assigned the wrong HPO ID.

More complex coordinations expectedly posed challenges to all evaluated NER models. For example, in the sentence “Contractures of 3rd and 4th digits,” the expected resolution is the doubly-disconnected named entities “Contractures of 3rd digit” and “Contractures of 4th digit.” The models encountered difficulty, likely due to the presence of the word “digit” in its plural form, which caused confusion in determining whether to resolve it as 2 separate entities or not. To provide a clearer overview of the types of normalization errors, we summarized their distribution in Table S4 in Multimedia Appendix 1. This table presents the proportion of each error category observed in the validation set, offering insights that may support future improvements in sentence-transformer–based normalizers.

### Limitations

Although the dataset used in this study was granted a waiver of HIPAA authorization for access to identifiable medical record information [42], our approach prioritizes privacy by enabling local deployment of open-source PLMs to ensure sensitive data remains secure. Unlike systems that rely on closed-source models such as GPT-4, our method leverages the open-source T5 family of PLMs. This makes the approach well-suited for use with confidential clinical datasets that are only accessible under restricted licenses. Additionally, it offers a cost-effective alternative to proprietary models.

Despite its reasonable performance, the model shows a tendency to favor the recognition of KEYFs, which were the primary focus of the shared task. This bias is likely caused by the class imbalance in the dataset and has not yet been explicitly addressed. Future work could explore strategies such as data augmentation or balanced sampling to mitigate this bias.

Although DiscHPO is specifically fine-tuned and evaluated on genetic phenotype findings, we hypothesize that the underlying generative and transformer-based framework is well-suited for clinical findings in other medical domains. The model architecture can be adapted to recognize NEs in diverse specialties like oncology, cardiology, or general medical NER. However, this study does not evaluate the system’s generalizability beyond datasets related to HPO, which remains a direction for future work. Furthermore, although the official competition evaluation was conducted using a gold-standard annotated corpus, evaluating the full system under more challenging conditions -such as noisy or unlabeled input- remains an important avenue for future work and would provide an indication of real-world applicability.

### Conclusions

This paper presents an overview of our DiscHPO, which is an NER and NEN pipeline capable of processing both continuous and discontinuous spans. We evaluated our model within the context of the BioCreative VIII - Track 3 challenge, focusing on extracting KEYF mentions of genetic phenotypes and normalizing them to the HPO. In the NER component, various T5 model variants were explored for KEYF extraction, and an additional experiment incorporated LoRA with extra-large models. From the extraction results, we found that optimizing Flan-T5-XL’s parameters using LoRA resulted in improved extraction outcomes for both continuous and discontinuous findings, leading to enhanced normalization performance.

Furthermore, improving normalization results can be achieved by resolving all disjoint mentions that share a common head, thereby avoiding the omission of relevant mentions. On the other hand, there may be cases in which extracting the exact mention, continuous or discontinuous, is not crucial for successful normalization. This is apparent in our observations of the normalization results, where several examples with partially extracted mentions still led to accurate normalization.

In future research, and inspired by Peng et al [14], there is a potential to enhance the accuracy of the NEN task by incorporating auxiliary components alongside the SBERT framework. For instance, combining semantic or lexical similarity measures (eg, TF-IDF or Cosine Similarity) with SBERT. Specifically, candidate generation could be performed in parallel using both SBERT and the similarity measure, with the aggregated set of candidates subsequently passed through a cross-encoder for final ranking. Moreover, we intend to conduct a comparative analysis between our DiscHPO model and an end-to-end NER and normalization model that shares contextualized embeddings for both tasks. This joint approach may improve performance by reducing error propagation across pipeline stages and enabling end-to-end optimization. Additionally, we aim to investigate the efficacy of autoregressive entity normalizers, as suggested in the work of De Cao et al [48].

## Data Availability

The datasets evaluated during this study are publicly available in the GitHub repository [49]. The source code for this study is available in a the GitHub repository [50].

## Appendix

Multimedia Appendix 1 Additional tables and figures.

## Abbreviations

BERT: bidirectional encoder representation from transformers
DiscNE: discontinuous named entity
EHR: electronic health record
HPO: human phenotype ontology
IE: information extraction
KEYF: key finding
LLM: large language model
LoRA: low-rank adaptation
NE: named entity
NEN: named entity normalization
NER: named entity recognition
NLP: natural language processing
NORMF: normal finding
PLM: pretrained language model
T5: text-to-text transfer transformer
