# Supplementary material for: Generative Models and Sentence Transformers for the Recognition and Normalization of Continuous and Discontinuous Phenotype Mentions: Model Development and Evaluation
Source: JMIR Med Inform. 2025 Nov 5;13:e68558. doi: 10.2196/68558 (PMC12631088; doi:10.2196/68558)
Supplement: Multimedia Appendix 1 [file medinform_v13i1e68558_app1.docx]

**Supplementary File**

**List of Revised Entity Spans**

Table S1 presents the identified inconsistencies in the textual spans, primarily resulting from inaccurate numerical offsets. We manually corrected these errors and provide a comprehensive list of the revised entity spans.

| **Instance No.** | **Inconsistency** | **Manual Revision** |
| --- | --- | --- |
| **Training Set** | | |
| 133 | NORMF: NA; NORMF: NA | NORMF: No frontal bossing; NORMF: Normal shape of the head |
| 200 | KEYF: right cheek enlarged right lip enlarged | KEYF: right cheek enlarged; KEYF: right lip enlarged |
| 327 | KEYF: rhizomelia) | KEYF: rhizomelia |
| 368 | KEYF: appear shortened; KEYF: toes appear shortened; NA | KEYF: toes 3 appear shortened; KEYF: toes 4 appear shortened; KEYF: toes 5 appear shortened |
| 1665 | KEYF: contractures of wrists; KEYF: contractures of wrists hands; KEYF: contractures of wrists, knees; KEYF: contractures of hips | 1665: KEYF: contractures of wrists; KEYF: contractures of hands; KEYF: contractures of knees; KEYF: contractures of hips |
| **Validation Set** | | |
| 152 | KEYF: Flat, nasal bridge | KEYF: Flat nasal bridge |
| 325 | KEYF: Long toes and | KEYF: Long toes |
| 365 | KEYF: Widely separated, teeth | KEYF: Widely separated teeth |

**Table S1.** Entity spans revisions identified during preprocessing.

**Optimal hyperparameter settings**

The following table outlines the optimal hyperparameter settings used in all T5 architectures throughout our NER experiments. The model’s weight is 786,432 parameters. The models were trained on a single NVIDIA A100 GPU with 40 GB of memory, employing the large versions of the models.

| **Hyperparameter** | **Value** |
| --- | --- |
| Sequence Length  Learning Rate  Optimiser  Batch size  Gradient accumulation steps  Epochs  Seed  LoRa r  LoRa α | 128  3e-4  Adam, epsilon=1e-8  8  16  20  45  16  1024 |

**Table S2.** NER hyperparameters' configurations

**Results on GCS+ dataset**

To evaluate the model's generalisability, we present DiscHPO's performance on the GCS+ dataset in comparison with two state-of-the-art baseline models.

| **Model** | **Precision** | **Recall** | **F1-Score** |
| --- | --- | --- | --- |
| PhenoTagger | 0.720 | 0.760 | 0.740 |
| PhenoRerank | **0.843** | 0.708 | 0.770 |
| PhenoGPT | 0.809 | **0.857** | **0.832** |
| DiscHPO | 0.8361 | 0.7115 | 0.7688 |

**Table S3.** Comparative performance of DiscHPO and baseline models on the GCS+ dataset.

In table S3, we show the performance results of DiscHPO on the GCS+ dataset. Our model outperformed PhenoTagger and achieved performance comparable to PhenoRerank. Which indicates that our model can be a lightweight alternative to weakly pre-training models with large number of instances. Notably, PhenoGPT surpassed all models, once again highlighting the strength of GPT-based approaches in this task, albeit with their trade-offs in terms of computational cost and privacy concerns. We observed some annotation-related inconsistencies and model errors. For instance, the dataset includes nested named entities, and the model failed to capture some nested cases such as "congenital lung cyst" along with "lung cyst". Nevertheless, fine-tuning still improved the model’s ability to extract simpler nested entities like "skin cancer" and "cancer", demonstrating its potential in handling such patterns. In terms of coordination ellipses cases, the dataset annotations often include the full unresolved phrase rather than marking the resolved forms. For example, the phrase “malformations of the kidney or collecting system” is annotated as-is. In contrast, our DiscHPO model tends to resolve it into two separate mentions— “malformations of the kidney” and “malformations of the collecting system”—reflecting its design to handle coordination ellipsis cases effectively.

**NEN error types**

| **Error Type** | **Count** | **percentage** |
| --- | --- | --- |
| Lexical similarity only | 28 | 18.06% |
| Overfitting to training data | 20 | 12.9% |
| Generalized concept predicted | 5 | 3.23% |
| Partially matched span | 37 | 23.87% |
| Over-extended span | 18 | 11.61% |
| Incorrectly extracted span | 39 | 25.16% |
| Inconsistent annotation | 8 | 5.16% |

**Table S4.** Distribution of NEN error types based on the validation set. Lexical similarity: the predicted concept is lexically similar but semantically incorrect. Overfitting to training: the model memorized training examples and failed to generalize. Generalized concept: returning a broad concept instead of a precise one. Partially matched span: only part of the entity mention is extracted. Over-extended span: including additional tokens. Incorrectly extracted span: when it does not correspond to any gold standard entity. Inconsistent annotation: ambiguity in the gold-standard annotations.
